# The Stethoscope Song

**Published:** 1887-08-13

**Authors:** Oliver Wendell Holmes


### The Stethoscope Song.

### A Professional Ballad.

There was a young man in Boston town,
He bought him a stethoscope nice and new,
All mounted and finished and polished down,
With an ivory cap and a stopper too.

It happened a spider within did crawl,
And spun him a web of ample size,
Wherein there chanced one day to fall
A couple of very imprudent flies.

The first was a bottle-fly, big and blue,
The second was smaller, and thin and long:
So there was a concert between the two,
Like an octave flute and a tavern gong

Now being from Paris but recently,
This fine young man would show his skill;
And so they gave him, his hand to try,
A hospital patient extremely ill.

Some said that his *liver* was short of *bile*,
And some that his *heart* was over size,
While some kept arguing all the while
He was crammed with *tubercles* up to his eyes.

This fine young man then up stepped he,
And all the doctors made a pause;
Said he,—The man must die, you see,
By the fifty-seventh of Louis's laws.

But since the case is a desperate one,
To explore his chest it may be well;
For if he should die, and it were not done,
You know the *autopsy* would not tell.

Then out his stethoscope he took,
And on it placed his curious ear;
*Mon Dieu!* said he, with a knowing look,
Why here is a sound that's mighty queer!

The *bourdonnement* is very clear,—
*Amphoric buzzing*, as I'm alive!
Five doctors took their turn to hear;
*Amphoric buzzing*, said all the five.

There's *empyema* beyond a doubt;
We'll plunge a *trocar* in his side.—
The diagnosis was made out,
They tapped the patient; so he died.

Now such as hate new-fashioned toys
Began to look extremely glum;
They said that *rattles* were made for boys,
And vowed that his *buzzing* was all a hum.

There was an old lady had long been sick,
And what was the matter none did know;
Her pulse was slow, though her tongue was quick;
To her our knowing youth must go.

So there the nice old lady sat,
With phials and boxes all in a row;
She asked the young doctor what he was at,
To thump her and tumble her ruffles so.

Now, when the stethoscope came out,
The flies began to buzz and whiz;—
Oh ho! the matter is clear, no doubt;
An *aneurism* there plainly is.

The *bruit de râpe* and the *bruit de scie*
And the *bruit de diable* are all combined;
How happy Bouillaud would be,
If he a case like this could find!

Now, when the neighbouring doctors found
A case so rare had been descried,
They every day her ribs did pound
In squads of twenty; so *she* died.

Then six young damsels, slight and frail,
Received this kind young doctor's cares;
They all were getting slim and pale,
And short of breath on mounting stairs.

They all made rhymes with "sighs" and "skies,"
And loathed their puddings and buttered rolls,
And dieted, much to their friends' surprise,
On pickles and pencils and chalk and coals.

So fast their little hearts did bound,
The frightened insects buzzed the more;
So over all their chests he found
The *râle sifflant*, and the *râle sonore*.

He shook his head; there's grave disease—
I greatly fear you all must die;
A slight *post mortem*, if you please,
Surviving friends would gratify.

The six young damsels wept aloud,
Which so prevailed on six young men,
That each his honest love avowed,
Whereat they all got well again.

This poor young man was all aghast;
The price of stethoscopes came down;
And so he was reduced at last
To practise in a country town.

The doctors being very sore,
A stethoscope they did devise,
That had a rammer to clear the bore,
With a knob at the end to kill the flies.

Now use your ears, all you that can,
But don't forget to mind your eyes,
Or you may be cheated, like this young man,
By a couple of silly, abnormal flies.

Oliver Wendell Holmes.

